# Impact of the severity of trauma on early retirement

**DOI:** 10.1186/1757-7241-20-S2-P26

**Published:** 2012-04-16

**Authors:** Michael Bilde Kuhlman, Nicolai Lohse, Anne Marie Sørensen, Claus Falck Larsen, Karl Bang Christensen, Jacob Steinmetz

**Affiliations:** 1 Rigshospitalet, Copenhagen University Hospital, Trauma Center, 3193, Blegdamsvej 9, DK-2100 København Ø, Denmark; 2 Global Stakeholder Engagement, Novo Nordisk A/S, Denmark; 3 Rigshospitalet, Copenhagen University Hospital, Department of Anaesthesia ANHOC, Blegdamsvej 9, 2100 København Ø, Denmark; 4 Rigshospitalet, Copenhagen University Hospital, Centre of Head and Orthopaedics, 2161, Blegdamsvej 9, DK-2100 København Ø, Denmark; 5 Department of Biostatistics, University of Copenhagen, Denmark

## Background

Injury Severity Score (ISS) classifies patients according to the degree of traumatic anatomical injuries and predicts subsequent risk of mortality. Trauma is known to cause loss of income. However, it is not yet known whether the ISS correlates with the risk of early retirement. Our aim was to assess the association between ISS and subsequent risk of voluntary early retirement- or disability pension.

## Methods

An observational cohort study based on prospectively collected data was conducted. We included patients aged 18-64 years who entered a level-one urban trauma centre during 1999-2007 and were still alive after three days of in-hospital care. Patients who were early retired (received voluntary early retirement- or disability pension) at the time of admission were excluded. Trauma data were linked to data on pension reception in the Danish Register-based Evaluation of Marginalization (DREAM) database. Patients were followed until new retirement (measured as being a new pension recipient), death or emigration. Risk of retirement according to ISS (low, ISS < 15 vs. high, ISS > 15) was assessed by Cox proportional hazards analysis, adjusted for gender and age.

## Results

In total, 1722 trauma patients were followed for a median of 6.2 years, interquartile range (IQR) 3.7-9.1. Of these, 76.2% were males, median age was 35.0 (IQR 25.4-46.5) years, and median ISS was 16 (IQR 9-25). Three hundred and twenty-two patients retired during follow-up. Patients with high ISS, compared to patients with low ISS, had an increased risk of early retirement, hazard ratio (HR) 2.50 (95% confidence interval 1.95-3.13; p < 0.001), adjusted HR 2.60 (2.05-3.30; p < 0.001).

## Conclusion

Severely injured patients with an ISS > 15 are at significantly increased risk of early retirement due to disability pension or voluntary early retirement.

